# Transforming systems of consumption and production for achieving the sustainable development goals: moving beyond efficiency

**DOI:** 10.1007/s11625-018-0582-1

**Published:** 2018-05-25

**Authors:** Magnus Bengtsson, Eva Alfredsson, Maurie Cohen, Sylvia Lorek, Patrick Schroeder

**Affiliations:** 1 Independent Researcher, Tokyo, Japan; 2 Royal Institute of Technology (KTH), Stockholm, Sweden; 3 New Jersey Institute of Technology, Newark, USA; 4 Sustainable Europe Research Institute, Cologne, Germany; 5 Institute for Development Studies, Brighton, UK

**Keywords:** Sustainable consumption and production, SDG implementation, Systemic approaches, Public policy

## Abstract

The United Nations formulated the sustainable development goals (SDGs) in 2015 as a comprehensive global policy framework for addressing the most pressing social and environmental challenges currently facing humanity. In this paper, we analyse SDG 12, which aims to “ensure sustainable consumption and production patterns.” Despite long-standing political recognition of this objective, and ample scientific evidence both on its importance and on the efficacy of various ways of promoting it, the SDGs do not provide clear goals or effective guidance on how to accomplish this urgently needed transformation. Drawing from the growing body of research on sustainable consumption and production (SCP), the paper identifies two dominant vantage points—one focused on promoting more efficient production methods and products (mainly through technological improvement and informed consumer choice) and the other stressing the need to consider also overall volumes of consumption, distributional issues, and related social and institutional changes. We label these two approaches *efficiency* and *systemic.* Research shows that while the efficiency approach contains essential elements of a transition to sustainability, it is by itself highly unlikely to bring about sustainable outcomes. Concomitantly, research also finds that volumes of consumption and production are closely associated with environmental impacts, indicating a need to curtail these volumes in ways that safeguard social sustainability, which is unlikely to be possible without a restructuring of existing socioeconomic arrangements. Analysing how these two perspectives are reflected in the SDGs framework, we find that in its current conception, it mainly relies on the efficiency approach. On the basis of this assessment, we conclude that the SDGs represent a partial and inadequate conceptualisation of SCP which will hamper implementation. Based on this determination, this paper provides some suggestions on how governments and other actors involved in SDGs operationalisation could more effectively pursue SCP from a systemic standpoint and use the transformation of systems of consumption and production as a lever for achieving multiple sustainability objectives.

## Transforming consumption and production—a key priority in the early 21st century

Humanity is facing a number of severe global crises in the early 21st century: growing ecological overshoot and worsening climate change, widespread deprivation and unmet human needs, and increasing inequality and social exclusion (Steffen et al. 2015; Roser and Ortiz-Ospina 2017; Milanovic 2016). Each of these situations has the potential to trigger social unrest, and concurrently threatens to undermine democracy and peace. They are also intricately linked with each other, and such interconnections are likely to amplify the related risks. These wicked problems need to be understood and addressed in a comprehensive fashion, reflecting their systemic nature.

The challenges mentioned above are, in various ways, closely associated with society’s consumption and production of goods and services. The ecological crisis—overuse of natural resources, pollution, and disruption of the planet’s natural process—is a direct reflection of what gets produced and consumed, and in what amounts. The humanitarian and social crises are to a large degree due to unequal access to energy and materials and opportunities to satisfy needs and wants. Dealing successfully with these dire threats thus requires a restructuring of how we produce and consume (e.g., Akenji et al. 2016).

The need for drastic changes in consumption and production is well reflected in the 2030 Agenda for Sustainable Development, both in the form of a commitment to make “fundamental changes in the way that our societies produce and consume goods and services”, and through having one of its seventeen sustainable development goals (SDGs) dedicated to ensuring sustainable consumption and production (SCP) (SDG 12) (Akenji and Bengtsson 2014). Elements of SCP, such as energy-efficiency improvements, can also be found as constituent elements of some of the other SDGs. However, the extent of this commitment remains highly ambivalent. Although a shift to SCP features prominently at the headline level of the new Agenda, the degree to which this agreement can be expected to drive transformative change toward a sustainable society depends on how specific targets and indicators are formulated and how they are interpreted by various implementing actors.

This paper presents a critical assessment of how the objectives of SCP are articulated in the 2030 Agenda. First, we show how SCP has emerged as a research field, involving over time an increasing range of academic specialisations, and provide a succinct characterisation of where this field stands today. We then examine the historical roots of the 2030 Agenda by showing how SCP has come to be reflected in mainstream policy discourse. Against this background, we then analyse how SCP is included in the SDGs framework and discuss how this framing compares to current research findings. In the final main section, drawing from preceding assessments, we provide some suggestions that have potential to underpin a major shift to SCP—thereby contributing to the overarching ambition of the 2030 Agenda—but that are not explicitly included in the current formulation of the SDGs.

## SCP research—dominant vantage points and recent advances

According to commonly deployed definitions (UNCED 1992; Norwegian Ministry of the Environment 1994), SCP seeks to achieve a good life for everyone within the constraints of the Earth’s biophysical capacity. Based on this broad objective, the concept has become associated with a wide variety of approaches including: consuming differently, using less resource-intensive products, moving from material products to immaterial services, energy conservation, sharing the use of products, and using higher quality products with longer lifespans (Lebel and Lorek 2008). A body of scientific research and a practitioner community has emerged around each of these approaches and the topic of SCP has progressively attracted researchers from an increasingly diverse array of academic disciplines. It is now a well-established interdisciplinary research field with regular academic conferences and an expanding number of publications. For instance, Google Scholar finds approximately 5980 publications on SCP published between 2008 and 2012, while for the following 5-year period (2013–2017), this number has grown to over 12,600.^1^ This section shows how the SCP research field has evolved, focusing on dominant vantage points at a meta-disciplinary level, and synthesises recent advances. The focus here is biased in favor of the consumption side, because ample attention has been devoted in recent years to distilling current understanding of production-oriented improvements (Pallaro and Subramanian 2015; Walls and Paquin 2015; Zamen 2015; Rajeev et al. 2017; Roy and Singh 2017; Merli et al. 2018; Govindan and Hasanagic 2018).

During the 1990s, research and governance pertaining to sustainable consumption mainly focused on household-energy efficiency and recycling. The emphasis was largely on trying to overcome the so-called consumption-sustainability dilemma through relative decoupling (O’Rourke and Lollo 2015). Publications and projects under the heading of sustainable consumption sought to promote best practices, often by providing single-product purchasing advice to consumers. As a result of this focus, one of the major elements of this discourse was to encourage consumers to take responsibility by buying “green” products—often guided by eco-labels or product guides. However, it soon became apparent that this approach would only bring about enhanced performance for selected products, for some consumers, or a few lifestyle groups; it was not a coherent and comprehensive approach for achieving sustainable consumption, which at the aggregate level is compatible with staying within planetary boundaries (see, e.g., Maniates 2001; Delmas and Burbano 2011). Instead, in the name of sustainable consumption, such greening interventions were—and still are—carefully calibrated efforts to manage the problems inherent in contemporary provisioning systems while not challenging their underlying social and environmental contradictions (Lorek and Vergragt 2015). The emphasis is on consuming differently—giving priority to products and services with lower associated environmental and social impacts compared to conventional alternatives. Such practices are intended to lead to changes within the current economic system and to spur sustainable growth (European Commission 2012). Unfortunately, rising demand for nominally “more sustainable” products tends to intensify energy and material throughput and hence to increase rather than decrease aggregate resource consumption (Owen 2011; Zehner 2012; Weidmann et al. 2015).

Concomitantly, approaching sustainable consumption solely from the point of view of economically motivated individuals or households has resulted in devotion of inadequate attention to the social and situational factors that influence both the intentions and actual behaviour of consumers (Steg and Vlek 2009). For example, theories of social practice offer established alternative perspectives focused not on personal attitudes but rather on the multitude of factors that shape actual behavioural routines (Hargreaves 2011; Huddart Kennedy et al. 2016; Strengers and Maller 2016).

Over the past two decades, it has, moreover, become increasingly apparent that product-based (and partly service-based) approaches that rely exclusively on technological innovation and efficiency improvement are necessary but insufficient for making significant progress toward sustainable consumption (Cohen et al. 2014; Davies and Doyle 2015; Huesemann and Huesemann 2011). Such interventions mostly amount to what has become known as a “weak” approach to sustainable consumption policy making (Fuchs and Lorek 2005; Fuchs 2013; Hobson 2013). Weak sustainable consumption strives to achieve *relative* improvements of product performance, but does not refer to *absolute* ecological limits, such as the need to rapidly curtail greenhouse gas (GHG) emissions at the global level to stabilize the climate (Steffen et al. 2015). Analyses of implementation pathways for the Paris agreement show that global GHG emissions need peak no later than 2020 and then fall rapidly. Given this urgency, Alfredsson et al (2018) argue that overall consumption needs to be reduced in order to achieve the Paris goal. One of the reasons being that consumption volumes are one of the main drivers of GHG-emissions, as well as of other environmental problems. A weak approach focuses, for example, on incrementally improving the fuel economy of automobiles, while a strong approach would explore a much wider range of options for cutting emissions from transportation—including other forms of transport as well as how the need for mobility might be transformed (Urry 2008; Schwartz 2015; Zipori and Cohen 2015). Considering the dire ecological and social challenges that we are facing, minor adjustments within the current system—mainly relying on “technological solutionism” and a product-based sustainable consumption approach—will not suffice to foster the changes needed to reverse current patterns of unsustainability. At best, we will under such circumstances manage to postpone pending ecological disasters and related socioeconomic crises.

Consequently, research in the field of sustainable consumption has begun to develop a variety of novel trajectories (Cohen et al. 2014; Reisch and Thøgersen 2015). Many academics—often working with civil society organisations or business groups—are increasingly getting involved in activities designed to enhance the sustainability of consumption and production, ranging from environmental science via the social sciences, economics, and business management (Wells 2013; Liu et al. 2017; Hoffman 2018). Both research and advocacy are being increasingly oriented around a systems approach, whereby the systems of technological innovation, industrial production, and mass consumption are understood to exhibit highly complex features, to have multiple feedback mechanisms, to be hard to grasp, to resist externally imposed changes, and to operate beyond the control of any single actor (Lebel and Lorek 2008, 2010). This expansive way of understanding the challenges of sustainable consumption ultimately entails changing the entire arrangement of investments, production, trade, consumption, and waste. It entails redesigning the economic system, associated infrastructures, dominant culture and lifestyles, as well as reforming institutions and reconfiguring power relationships (Vergragt et al. 2014). Power asymmetries have been found to play an especially central role in creating structural barriers to sustainable consumption and in delimiting opportunities for intervention (Fuchs et al. 2016). Consideration of power relations results in the need to address institutions that shape production and consumption systems, including reorganising key aspects of the financial sector (Fullerton 2012; Røpke 2017). When applying a systems perspective to SCP, governance and governing become central foci for attention (Tukker 2008; Isenhour 2016; Keller et al. 2016; Evans et al. 2017).

Lebel (2004) describes a system of sustainable consumption as “a system that links environmental goods and services, individuals, households, organisations, and states through linkages in which energy and materials are transformed, utility is derived, and relationships (for example, transactions of money or information and exercise of influence and social control) take place.” Understood in these terms, a system of sustainable consumption and production (SSCP) is one in which the transformation of energy and materials maintains or improves human well-being (or utility) without irreversibly depleting or degrading environmental resources. A systems perspective allows consideration of alternative ways of governing the provisioning of energy and materials not just at particular points of activity, as in markets or through trade agreements, but along supply and waste chains. Such a perspective also brings issues of fairness and social justice to the fore (Lebel and Lorek 2008).

To summarise, we identify two distinct vantage points in the SCP literature—one focused on promoting more efficient production methods and products (mainly through technological improvement and informed consumer choice) and the other stressing the need to consider also overall volumes of consumption, distributional issues, and related social and institutional reforms. While these two perspectives may seem to be in conflict with each other, we regard them mainly as complementary. More specifically, we see the latter perspective—which we in this paper will call *systemic*—as an evolution and expansion of the former, which we here label *efficiency.* A *systemic* approach to SCP thus recognises the benefits that enhanced *efficiency* can bring, the need for technological changes, and the value of having well-informed consumers, but it stresses at the same time the necessity of a range of additional measures for achieving sustainable consumption and production patterns.

## SCP in international sustainability agreements—a look in the rear-view mirror

During the decades following World War II, the conventional tendency was to attribute nascent environmental problems at the international scale to rapid population growth. This view stemmed from the fact that improvements in public health had combined with other sources of social and economic change to sharply shift demographic patterns throughout large parts of Asia, Africa, and Latin America (Bandarage 1997; Connelly 2008; Ehrlich 1971; Meadows et al. 1972). The conceptions that emanated from these circumstances ascribed responsibility for the overexploitation of resources to the developing countries.

Publication of the Brundtland Commission’s report, *Our Common Future*, in 1987 marked the early emergence of a distinctly new and insurgent perspective which introduced affluence and consumption patterns as important drivers of environmental problems and put the challenge of sustainable development on the international policy agenda (WCED 1987).^2^ The authors provocatively wrote that

[l]iving standards that go beyond the basic minimum are sustainable only if consumption standards everywhere have regard for long-term sustainability. Yet many of us live beyond the world’s ecological means, for instance in our patterns of energy use. Perceived needs are socially and culturally determined, and sustainable development requires the promotion of values that encourage consumption standards that are within the bounds of the ecological possible and to which all can reasonably aspire.

Implicit in this critique was recognition that the lifestyle practices prevalent in high-consuming nations were responsible for outsized resource demand as well as exceeding the capacity of biophysical sinks to assimilate waste byproducts (Dauvergne 2010).

The issue of sustainable consumption, however, first received careful and publicly prominent consideration at the United Nations Conference on Environment and Development (UNCED) held in Rio de Janeiro in 1992. Agenda 21—the major outcome document of UNCED—contains a chapter entitled “Changing Consumption Patterns,” which states that “All countries should strive to promote sustainable consumption patterns [and]…[d]eveloped countries should take the lead” (United Nations 1992). However, the text was decidedly ambivalent about the role of consumption as a driver of excessive resource appropriation due to resistance on the part of several key countries to even consider the adverse social and environmental implications of economic growth (Redclift 1996; Cohen 2001).

During the years following the Rio Summit, the Nordic Council and its constituent countries played an important role in developing initial policy prescriptions to encourage less resource-intensive modes of consumption, though mainly in ways that would not threaten economic growth or risk triggering social or economic instability (see, e.g., Nordic Council of Ministers 1995). For instance, at a symposium held in Oslo during the mid-1990s sustainable consumption was defined as “the use of services and related products which respond to basic needs and bring a better quality of life while minimizing the use of natural resources and toxic materials as well as emissions of waste and pollutants over the life cycle of the service or product so as not to jeopardize the needs of future generations” (Nordic Council of Ministers 1995).

Further assisting the agenda-setting process was a highly influential work program conceived and implemented at the time by the Organisation for Economic Co-operation and Development (OECD 1997, 1997) and a joint project of the National Academy of Sciences in the United States and the Royal Society of London (1997). It was also during this period that the first tranches of research funding were allocated, first by the European Science Foundation and then various national science councils of member countries.

Somewhat separately, different communities of practice were engaged in efforts to develop scientific and policy capacity around the complementary notion of “sustainable production.” This focus area built on much more deeply established expertise and experience at the interface between industrial engineering and environmental science and was centered on clean manufacturing, toxics reduction, byproduct exchange, and economic circularity and sought to refine several emergent methodological techniques including life cycle analysis, material flow analysis, and environmentally extended input–output analysis (Frosch and Gallopoulos 1989; Ehrenfeld 2004). A significant step forward in amalgamating these modes into an integrated conceptual framework started with formal establishment of the field of industrial ecology in 2001 and it gradually became common during this time for practitioners, policy makers, and others to conjoin sustainable consumption with the pursuit of sustainable production. While such a fusing was in some respects perfectly sensible and prudent, this move was also driven by political and institutional rationales. In particular, the more technical interventions associated with production-focused strategies made it possible to sublimate, and at times completely subsume, the insurgent ideas advanced by proponents of sustainable consumption (Murphy 2001; Cohen and Howard 2006). At the same time, the different disciplinary and epistemic foundations of the production and consumption sides of this undertaking have made it extremely difficult to develop fully meaningful and legitimate linkages (cf. Tukker et al. 2010; Mont and Heiskanen 2014; Moreau et al. 2017).

Such was the status of the uneven relationship during the period leading up to the World Summit on Sustainable Development in Johannesburg in 2002 (Barber 2003; Fuchs and Lorek 2005), but this event nonetheless gave rise to a call by national governments to establish a 10-year framework of programs (10YFP) on SCP. The initiative was advanced during subsequent years under the banner of the so-called Marrakesh Process which was led by the United Nations Environment Program (UNEP) in collaboration with a network of nongovernmental organisations and independent researchers (Church and Lorek 2007; Clark 2007). After a decade of halting progress on SCP, the 10YFP was adopted at the United Nations Conference on Sustainable Development (Rio+20) in 2012, and in more recent years, sustainable consumption (and production) has become a progressively visible focal point for a growing number of international initiatives. For instance, as noted above, the 2030 Agenda for Sustainable Development contains a headline goal devoted to “ensuring sustainable consumption and production patterns.” The significance of a shift to SCP is also recognised in the 2015 Paris Agreement on Climate Change, which states that “sustainable lifestyles and sustainable patterns of consumption and production, with developed country parties taking the lead, play an important role in addressing climate change” (UNFCCC 2015; see also Reisch et al. 2016). More ambitiously, the role of SCP is highlighted in the Convention on Biological Diversity, where the 2010 Aichi Targets include a focus on SCP, where signatories agree that “[b]y 2020, at the latest, Governments, business and stakeholders at all levels have taken steps to achieve or have implemented plans for sustainable production and consumption and have kept the impacts of use of natural resources well within safe ecological limits” (CBD 2010).^3^

To summarise, SCP has been part of the international policy discourse for more than four decades, but during this time, the uptake has not been entirely smooth and has tended to be biased toward relatively weak measures. Despite some early statements recognising the need for changes in lifestyles and consumption patterns, and arguably strong formulations in a number of international agreements, there has been a reluctance to act on these insights and a tendency to focus on technological solutions and mainly on the production side. The *efficiency* approach identified in the preceding section has, despite ample evidence of its limited efficacy, been the dominant paradigm for policy action, while the broader *systemic* approach has had very limited traction.

## SCP in the 2030 Agenda for Sustainable Development—a constructive critique

The 2030 Agenda for Sustainable Development acknowledges the important role of SCP in its introductory section, includes this objective as one of the seventeen sustainable development goals (SDGs), and has SCP-related targets as part of several other SDGs. The SCP goal (SDG 12), which aims to “[e]nsure sustainable consumption and production patterns” by 2030, consists of eleven targets (three of which are intended as means of implementation), supported by thirteen indicators. In a general sense, SCP thus features prominently in the 2030 Agenda. However, the extent to which this recognition can underpin the transformative changes explicitly aimed for in the agenda depends partly on how SCP is defined and how it is elaborated into specific targets and indicators.

In this section, we, therefore, analyse how SCP is conceptualized in the 2030 Agenda, focusing mainly on its overall framing and the structure of SDG 12, while also briefly discussing how SCP is referred to in the other SDGs. We base this analysis on two complementary frameworks. First, we use the distinction made in preceding sections between a narrow approach to SCP, which relies primarily on enhanced *efficiency*, and a more comprehensive approach which stresses the need also for *systemic* change. Second, we use the so-called “input–activity–output–outcome–impact” framework commonly employed by development practitioners for project monitoring and evaluation (W.K. Kellogg Foundation 2004). This approach distinguishes between targets/indicators formulated at different stages of a causal or temporal chain. It is relevant to our analysis, since it helps us see whether targets and indicators mainly focus on providing means (input, activities, and output) or on identifying higher order objectives (outcomes and impacts) and it can inform a discussion about the consistency between means and objectives.

Our analysis aims to reveal what aspects of SCP are well covered by the 2030 Agenda and what aspects are ignored or only partly addressed. Especially, the latter—the identification of blind spots of the agenda—is of great practical relevance, since it highlights areas, where implementing agents (including both governments and various stakeholders) need to make efforts to “fill the gaps” beyond what is explicitly agreed. In the next section of this paper “Facilitating a transformation to SCP—toward more systemic approaches” section, drawing from the extensive SCP literature, we provide some suggestions in this direction.

### The overall framing of sustainable consumption and production

While the 2030 Agenda does not include an explicit definition of SCP, the introductory sections of the agreement provide some indications. The overall level of ambition regarding SCP is reflected in a commitment to “fundamental changes in the way that our societies produce and consume goods and services.” From this statement, it is clear that the agreement recognises the need for profound changes in patterns of consumption as well as in production. However, the nature of those changes is not outlined in any greater detail.

The preamble of the agenda identifies SCP as key to protecting the planet from degradation, so that it can support the needs of present and future generations. The notion of SCP is also mentioned in the overall vision: “A world in which consumption and production patterns and use of all natural resources—from air to land, from rivers, lakes and aquifers to oceans and seas—are sustainable.” Focus thus seems to be on maintaining the natural resource base needed for continued human civilization. However, the text also envisages a world “in which humanity lives in harmony with nature and in which wildlife and other living species are protected,” which indicates a recognition that nature has a value in its own right, beyond its value as a resource for humans.

As seen above, the agenda makes an explicit link between SCP and inter-generational equity, but the text is less clear concerning the need to ensure a more equitable distribution of consumption opportunities also in the current generation. Although the agreement points out the need for wealth sharing, and for addressing income inequality, it does so in relation with the notion of “inclusive growth” and without any reference to redistribution. The agenda thus apparently seeks to rebalance consumption opportunities through growing overall levels of consumption rather than through reallocation of consumptive opportunities. This awkward formulation reflects an implicit assumption that there is ecological space available for such increases in aggregate consumption.

### SDG 12—ensuring sustainable consumption and production patterns

The first target of SDG 12 (12.1) is to implement the 10-Year Framework of Programs on SCP (10YFP), which aims to accelerate a shift toward SCP in both developed and developing countries. This initiative is coordinated by UNEP and originally consisted of six thematic programs: sustainable public procurement (SPP), consumer information for SCP (CI-SCP), sustainable tourism program (STP), sustainable lifestyles and education (SLE), sustainable buildings and construction (SBC), and sustainable food systems (SFS) (the SPP program was later turned into a “large scale programmatic area”). The indicator for this target is the number of countries with national SCP action plans.

It is hard to assess the potential efficacy of this target. It can be seen merely as a reconfirmation of an existing international initiative, and as such, the target adds little impetus to a transition to SCP. What the target can achieve depends on how the 10YFP as such is designed and managed, what resources the programs have at their disposal, to what degree they can secure strong commitment from a wide range of actors, and other factors.

The stated objective of the 10YFP is to contribute to enhanced *efficiency* and decoupling, to be achieved mainly through knowledge sharing and capacity building. The objective is thus to reduce negative impacts in a relative sense, but there is no clear recognition of a need to limit or reduce overall volumes of consumption in line with a broader *systemic* approach.

The indicator is at the level of activity (the drafting and adoption of national plans), which leaves open both the question of what input (resources) is needed in order for these activities to be successful and what higher order changes (outcomes and impacts) are expected. The indicator is also based on the implicit assumption that national SCP action plans will actually be rigorously constructed and effectively implemented, or at least have significant influence on the actions taken by governments and various stakeholders. Finally, the indicator seems to assume that separate national SCP action plans are the preferable way to integrate SCP into the work of governments. In reality, other approaches, such as integration of SCP objectives into mainstream economic development planning processes or into sectoral plans and policy frameworks may be equally or more effective.

Target 12.2, which aims to “achieve the sustainable management and efficient use of natural resources,” reflects the need to temper human society’s impact on natural systems. As such, it is a critical element of achieving a sustainable society. It is formulated at the level of Impact. The target will be monitored with the help of six indicators, measuring countries’ overall consumption of natural resources, both in absolute terms, on a per-capita basis and per unit of gross domestic product (GDP). The indicators further measure resource consumption based on two different accounting principles: the volumes of resources extracted and used in each country (domestic material consumption), and the amounts of resources needed to support a country’s final consumption of goods and services, including the impacts of trade (material footprint).

At a highly aggregated level, these indicators can be used for monitoring changes in national economies’ pressure on the planet—from different perspectives. The two indicators, where resource consumption is divided by GDP, can be regarded as measures of the *efficiency* of national economies, but as relative metrics, they do not show whether the overall pressure on planetary systems is increasing or decreasing. This aspect is, however, covered by the absolute measures of resource use. The per-capita-based indicators can show how countries differ in terms of citizens’ average material standard, especially the footprint indicator. As such, these indicators are relevant for monitoring inequality (Steinmann et al. 2017). Taken together, the six indicators provide a good basis for *systemic* actions.

However, the wording of the target raises some questions. While the management of natural resources needs to be sustainable, the use (consumption) of such resources only needs to be efficient. This gives the impression that resource extraction and consumption are not directly connected; as long as natural resources are sustainably managed, there appears to be no need to consider the sustainability of consumption as such. This ambiguity may reflect a reluctance to commit explicitly to sustainable consumption. In addition, although the four indicators can provide a robust quantification of resource consumption, the target does not specify what levels are to be achieved by 2030—not even the desired direction of change. In consequence, many implementing agents are likely to be wondering how to set quantitative objectives for themselves in relation with this SDG target and which of the four indicators to pay most attention to. Finally, the target provides no guidance on what activities and short-term output should be considered for achieving the envisaged Impact.

In sum, target 12.2 is critical for ensuring the ecological sustainability of the 2030 Agenda and curbing resource consumption is key to achieving a number of other goals, especially SDGs 13 (climate change), 14 (life below water), and 15 (life on land). These “green” targets, in turn, are essential for realising key social objectives, including human health, food security, and poverty eradication. However, as mentioned above, a number of key questions on how to operationalise the 12.2 target are left open.

The following three targets—12.3, 12.4, and 12.5—are all concerned with waste and follow a similar logic in trying to reduce the negative environmental impacts of consumption and production systems, enhancing their *efficiency.* However, the targets do not indicate any need to change the structure of such systems in any significant way or to modify the volume or composition of consumption. Let us review each of these three targets in turn.

First, target 12.3 seeks to halve per-capita global food waste at the retail and consumer levels and to reduce food losses along production and supply chains, including post-harvest losses. Its indicator is the global food-loss index currently being developed by the United Nations Food and Agriculture Organisation (FAO). It is an outcome target that could help address the environmental impacts associated with the treatment of food waste, but where the wider impacts occur is not fully clarified. For example, it is not evident how the envisaged reduction in food waste would affect the overall volume of food production and its environmental impacts or to what extent it could contribute to hunger relief. This is the only SDG12 target that has a quantified reduction objective. However, although the target seems to aim for cutting food waste by half in absolute terms, the food-loss index measures waste in relative terms as percentage of food input (FAO 2017). Based on this characterization, we consider this target as also aiming mainly for enhanced *efficiency* of agri-food systems.

Second, target 12.4 seeks to achieve, by 2020, the environmentally sound management of chemicals and all waste. For chemicals, this is just a reconfirmation of what governments and stakeholders committed to already in 2006 with the adoption of the Strategic Approach to International Chemicals Management (SAICM) policy framework, but for waste management, it is a new commitment. It is an Impact target that will be very challenging to achieve, even after 2020. Moreover, it will be difficult to monitor progress, since there is no agreed definition of “environmentally sound management.” The indicators measure progress in terms of the number of parties (i.e., national governments) that transmit information, the amount of hazardous waste generated per capita, and how such waste is treated. The first of these indicators refers to countries’ compliance with previously agreed treaties on chemicals management and thus adds nothing new. Monitoring the volumes of hazardous waste generated and how that waste is treated is clearly important but there is no indicator tracking the treatment of non-hazardous waste, which is also causing serious health and environmental impacts.

Finally, target 12.5 aims to substantially reduce waste generation through prevention, reduction, recycling and reuse. It is similar to the target on curtailing food waste but with a much broader scope. Although the target mentions four different approaches to waste reduction, the indicator only measures recycling (national recycling rate and amount of waste recycled), which is often the least preferable option from an environmental perspective, as indicated, for example, in the European Union waste hierarchy. This preference is in line with a technology-focused efficiency *approach* and reflects a weak commitment to a broader *systemic* strategy.

The following three targets (12.6, 12.7, and 12.8) are all about encouraging action from various actors—private companies, public sector, and individual citizens.

Target 12.6 is intended to encourage companies, especially large and transnational companies, to adopt sustainable practices and to integrate sustainability information into their reporting cycle. It is an activity target that seeks to improve *efficiency*, presumably mainly of production systems. It is phrased in a vague fashion, using the word “encourage” rather than stronger alternatives, such as “require.” The indicator is the number of companies that publish sustainability reports, hence focusing on the latter and much easier part of the target—to provide information. Increasing the transparency of how companies address sustainability can be a step toward improved practices, but there is little evidence that this measure by itself will have significant impact.

Target 12.7 promotes public procurement practices that are sustainable, in accordance with national policies and priorities. It is an activity target that could have significant impact given that governments are major purchasers of goods and services. However, just like the preceding target, is vaguely phrased, aiming only to promote better practices. This target is linked to the 10YFP which has a specific program on sustainable public procurement. The indicator that measures the number of countries implementing sustainable public procurement policies and action plans is not very meaningful, because the metric says nothing about the level of ambition of such plans or the extent to which they are implemented.

Target 12.8 is designed to ensure that people everywhere have the relevant information and awareness for sustainable development and lifestyles in harmony with nature. The language of this target is stronger than the previous two targets (to ensure) and it addresses citizens. The target, if seriously implemented by governments, could contribute to achieving SCP and sustainable lifestyles. However, as research has shown, providing information and raising awareness on their own will not be sufficient to achieve the required transformation of currently unsustainable consumption and production patterns. Like the previous target, it is closely linked with one of the 10YFP programs—the one on sustainable lifestyles and education. It is also linked to target 4.7 under the SDG on Quality Education.

Both target 12.7 and 12.8 address themes also covered by the 10YFP (target 12.1). They can, therefore, be seen as attempts to put more weight behind these themes and to encourage broader uptake of 10YFP activities by national governments.

The final three targets of the SCP goal (12.a, 12.b, and 12.c) are designated as “means of implementation,” which presumably means that they are intended to help achieving the other targets.

Target 12.a is to support developing countries to strengthen their scientific and technological capacity to move toward more sustainable patterns of consumption and production. This target has a strong focus on technological solutions and *efficiency*. Progress will be measured by the amount of support provided to developing countries on research and development for SCP and green technologies. This is the only SDG12 indicator clearly monitoring implementation at the input level, although the level and kind of support to be provided are not specified. It is unclear how much additional support this target will mobilise beyond what wealthy countries are already providing through bilateral and multilateral channels.

While the capacity strengthening called for in this target can be beneficial for a shift to SCP, it will be challenging to ensure that it actually contributes to more sustainable outcomes. It is somewhat ironic that wealthy countries with superior scientific and technological capacity have far higher per-capita environmental impacts than developing nations. This indicates that technological prowess as such does not ensure sustainable consumption and production patterns, but can in fact have the opposite effect.

Target 12.b is to develop and implement tools to monitor sustainable development impacts for sustainable tourism that creates jobs and promotes local culture and products. This target is very narrow, with its focus on tools for monitoring impacts in a specific economic sector. It is, therefore, hard to see how it could be effective as a means of implementation across the other SDG12 targets. The target is strongly related to the 10YFP (target 12.1), and may be redundant, since the 10YFP program on sustainable tourism is likely to develop and promote tools of this kind. The indicator for the target is the number of sustainable tourism strategies or policies—an output that is difficult to measure in a meaningful way and for which it is also hard to assess the potential impact.

Target 12.c is to rationalise fossil-fuel subsidies, but the target text contains a number of caveats, such as a reference only to “inefficient” subsidies, which makes it possible for governments to maintain such subsidies if they so wish. The target is a reconfirmation of what G20 countries committed to already in 2009. Reduced subsidies can be regarded as an outcome level target, but it could also be seen as an input, since it would shift incentives for a range of activities. It would provide incentives for enhanced *efficiency* and for shifting consumption away from fossil-fuel-intensive goods and services. However, unless overall consumption volumes are also addressed, in line with a *systemic* approach, this could have undesirable side-effects, such as worsening environmental impacts caused by increasing production of bio-based fuels. One indicator measures fossil-fuel subsidies per unit of GDP, which means that total amounts of subsidies can remain constant, or even increase, although the indicator value decreases. The other indicator measures fossil-fuel subsidies as a proportion of total national expenditure on such fuels.

None of the three targets designated as means of implementation are time-bound, which is inconsistent juxtaposed to the urgency otherwise undergirding the 2030 Agenda. Given their role as enablers for other targets, it would make sense to ensure that good progress is made in these areas in the early phases of implementation.

To summarise this appraisal of SDG 12, 3 of the 11 targets mainly confirm earlier agreements or recognise existing initiatives (the 10YFP, SAICM on sound chemicals management, and the G20 agreement on fossil-fuel subsidies). These targets thus offer little new in terms of clearer commitments or additional resources. A partial exception is the target on chemicals, which also aims to achieve environmentally sound management of all waste. Three of the remaining eight targets address themes that are also focus areas of the 10YFP (sustainable public procurement, sustainable lifestyles and education, and sustainable tourism). In that sense, they mainly emphasise the significance of already ongoing efforts. Of the remaining five targets, one is about capacity building (in developing countries) and one on increased disclosure of information (by private companies), but the wording of these two targets is vague and the efficacy of these measures is uncertain. Four targets, including the one on chemicals safety and waste management mentioned earlier, address different aspects of society’s resource use and they provide new commitments beyond existing international agreements. Three of these targets deal with waste—the downstream of the economy—while only one target addresses the natural resources that enter the economy on the upstream side. Taken together, these targets could provide a basis for addressing society’s throughput of materials in a *systemic* fashion, but, as mentioned above, there is a bias toward end-of-pipe solutions, such as recycling, and an emphasis on improvements in relative terms (*efficiency*) rather than as absolute reduction in material throughput. There is a risk that governments will assign the responsibility for leading the implementation of these targets to ministries in charge of environmental protection, which in most countries have limited mandates that constrain their ability to address the issues of resource consumption and waste generation in a *systemic* way.

Most targets envisage intermediary outputs or outcomes, while only one target has a clear focus on providing input. In addition, the input level target is phrased in such a way that it will be challenging to demonstrate whether any additional resources, beyond already intended contributions, have been provided. Furthermore, some of the outcome targets (on information and knowledge provision) seem to rely on simplistic assumptions concerning their contribution to higher order impacts.

Finally, concerning indicators, for several of the SDG 12 targets, the agreed metrics cover only a certain aspect of the actual target. In some cases, such as with waste reduction and sound waste management, the indicators do not even reflect the most salient aspects of the targets as such and are thus not even useful as proxies.

### SCP in other sustainable development goals

In addition to SDG 12, SCP objectives are included as targets under a number of other goals. In SDG 8, “Promote sustained, inclusive and sustainable economic growth, full and productive employment and decent work for all”, target 8.4 is focused on improving global resource efficiency in *consumption and production* and endeavouring to decouple economic growth from environmental degradation. Whether the decoupling referred to is relative or absolute is not explicit. Target 8.4 also mentions the 10 YFP on SCP (Target 12.1) and is to be monitored with the same set of indicators as Target 12.2. The reference to SCP in Goal 8 acknowledges its role in ensuring environmental objectives and as a counterbalance to Target 8.1, which aims to bolster economic growth—to “sustain per-capita economic growth in accordance with national circumstances and, in particular, at least 7% gross domestic product growth per annum in the least developed countries”. It is important to highlight that, based on historical data pertaining to economic growth and material consumption, Target 8.1 is in sharp conflict with Target 12.2. Unless economic growth is drastically decoupled from resource consumption sustainable management of resources will not be possible. However, despite a great number of related policy initiatives over long time, no such resource decoupling has been achieved to date at the global level. This points to the existence of a serious goal conflict within the SDG framework, which requires more scrutiny and debate (see also Spangenberg 2016).

Although SCP is not mentioned explicitly in other SDGs, core elements of SCP are included in a number of other goals. For instance, Target 7.3 aims to double the global rate of improvement in energy efficiency by 2030. Improving energy efficiency in housing and mobility is at the core of many SCP initiatives worldwide, including the 10 YFP, but such improvements may not translate into corresponding decreases in environment pressure. Under SDG 6 on clean water and sanitation, SCP approaches will be required to achieve Targets 6.3 (reducing water pollution and hazardous chemicals) and 6.4 (increase water-use efficiency). SCP approaches are relevant for Goal 11 on sustainable cities and communities, in particular 11.6 which aims at improving municipal waste management (although not reducing overall amounts of waste) and improving ambient air quality in cities.

### Working systemically with the existing SDG 12 targets

As discussed above, the SDG 12 mainly represents an *efficiency* approach to SCP and, as such, its overall efficacy can be doubted. Even so, it contains targets that can be useful starting points for *systemic* actions—especially those that focus on society’s material throughput. In the following, we provide a few examples of what his could entail.

SDG 12.2 aims to achieve “sustainable management and efficient use of natural resources” and is a target of strategic importance for ecological sustainability. However, the target does not indicate how the volumes of resource use should change to become more sustainable—whether a stabilisation at current levels is sufficient or whether an overall reduction is considered necessary. Determining what levels of resource consumption can be considered sustainable is admittedly very complex and more analysis is needed. Even so, there are strong indications that current levels of material throughput are already too high and that an overall reduction should be pursued (e.g., Wackernagel et al. 2017).

SDG 12.3 aims to reduce per-capita food waste by half. This could potentially have multiple sustainability benefits, including improved food safety and nutritional status for low-income groups and reduced environmental impacts, but a narrow “end-of-pipe” focus may not fully realise such benefits. A systemic approach would require carefully analysis of the multiple factors that influence how agri-food systems are structured, how they operate and evolve, and why they generate such large amounts of surplus/discarded food. A systems approach would seek to identify and address such underlying factors. To reduce food waste from supply chains, such analyses may for example identify a need for localised food systems with shorter geographical distance between sites of production and consumption and fewer intermediary transactions. Another example could be to review how agricultural subsidies lead to artificially low food prices and surplus amounts of food loss, and to reform such economic incentives.

A similar systems perspective is needed when working with the other two waste-related targets (12.4 on chemicals and waste and 12.5 on reuse, waste reduction, and recycling). A world free from chemical hazards and one where material flows are predominantly circular cannot be achieved by focusing on the downstream material discharges of systems of consumption and production. As has long been recognised within the research community, moving toward these objectives will require changes in, for example, product design (Spangenberg et al. 2010), business models (Boons and Lüdeke-Freund 2013; Charter et al. 2008), and rules on which chemical substances can be lawfully produced (Lilienblum et al. 2008).

Working with the linkages between the SCP targets and other goals of the 2030 Agenda is also of critical importance. This includes considering how SCP targets may have the potential to reduce trade-offs between goals. For example, the SDG Interactions report by the International Council of Science (ICSU 2017) identified possible conflicts between SDG 2 and SDG 15 as “increased agricultural production and productivity, if not sustainable, can result in deforestation and land degradation, jeopardizing long-term food security. A careful balance is needed between achieving food for all and conserving and restoring ecosystems.” The SCP Target 12.3 focused on substantially reducing food waste and food losses by 2030 is an important aspiration that can reduce this trade-off and achieve careful balance between providing food for all and protecting ecosystems.

## Facilitating a transformation to SCP—toward more systemic approaches

Despite the clear centrality of SCP with respect to the global challenge of sustainability, “SCP in the 2030 Agenda for Sustainable Development—a constructive critique” section demonstrated that there are serious shortcomings in the way SCP is currently conceptualized in the SDG framework—focused mainly on efficiency improvements and relying heavily on knowledge sharing as a mechanism for social change. This represents an approach that SCP research has found to be inadequate but that has for a long time been dominant in policy discourse, including international agreements on sustainability “SCP research—dominant vantage points and recent advances” and “SCP in international sustainability agreements—a look in the rear-view mirror” sections. Based on this analysis, this section outlines a number of action areas for policy makers and others to consider in the context of SDGs implementation. These suggestions reflect on the necessities and possibilities for *systemic* approaches as are emerging in research on consumption and production.

### Driving transformation toward SCP: beyond the SDG 12 targets

The SCP agenda is very broad and complex and impossible to comprehensively capture in a few brief target statements and a handful of performance indicators. It is thus only natural that there are many aspects of SCP that are not represented in SDG 12. However, as discussed above, the agreed targets and indicators have a certain bias and leave out some important dimensions—something that will hamper implementation.

Most basically, the current formulation limits itself to the environmental dimensions of sustainability, which is a severe misrepresentation of what the SDGs intend to enable: an equitable human flourishing in a shared biosphere. To better create synergies between human well-being and ecological sustainability, rather than conflicts and trade-offs, a number of reform areas are proposed in the rapidly growing SCP literature. Many of these linkages could additionally help to connect SCP to other goals in the SDG framework. We highlight below a few action areas that are deemed to be particularly important for a shift to SCP, but that are absent from SDG 12.

#### Curtailed paid labour

Innovation and the adoption of more sophisticated ways of organising production systems have greatly enhanced labour productivity and continue to do so (Sanne 1992; Schor 1998, 2005; Devetter and Rousseau 2011; Kallis et al. 2013; Nässén and Larsson 2015). Current volumes of production are pressing against ecological limits, while at the same time, many people in wealthy countries (as well as in the Global South) experience overwork and other nations struggle with unemployment and underemployment (Maniates 2010). These circumstances suggest that more explicit effort should be devoted to using improvements in labour productivity to free up time for other activities. Shorter working hours could have a number of benefits linked with objectives of the other SDGs, for example, improved health and a revitalised civil society (Knight et al. 2013; Rosnick and Weisbrot 2007).

#### Good quality public services

Public services that are accessible and affordable to all can support inclusive well-being while moderating the need for private consumption and ownership, resulting in lower environmental pressure. Libraries, parks, swimming pools, open-air gyms, community centres, repair fairs, and public transportation systems are just a few examples of such facilities that can enable less consumptive lifestyles. Community-based products and various kinds of sharing initiatives also belong to this category (McLaren and Agyeman 2015; Gorenflo et al. 2018). There are elements of this in SDG 13 on sustainable cities, but there is room to explore much more directly how public infrastructure and services can help reinforce SCP objectives. At the national level, robust welfare systems with free education and universal health insurance can contribute to reduced inequality, elitism, and status-driven consumption. Inequality is an SDG goal with strong linkages to SCP, where implementing agents can seek to generate synergies between social progress and ecological sustainability.

#### Support for cooperatives, worker-owned companies, community ownership and small-scale businesses rooted in communities

Alternatives to profit-driven enterprises seek to generate benefits for people directly involved rather than for anonymous owners and investors (Bocken and Short 2016). Such models need to be carefully contrasted with for-profit enterprises, in part, because they can provide multiple values in terms of livelihoods, strengthened community bonds and trust, proximity to members/consumers, and reduced need for motorised transport, and often an ability to take greater responsibility for environmental impacts (Gelbmann and Hammerl 2015). A strong argument can be made for government support of these kinds of initiatives and their supporting institutions and this is occurring in a number of locales (Jones Austin 2014; Kerr 2015).

#### Spurring health benefits

There are ample opportunities to create synergies between SCP objectives and the SDG on human health. Healthy, active lifestyles, including health-promoting diets, have the potential to also be less resource intensive and to have lower environmental impact.

### Moving sustainable consumption up the political agenda

The one-sided representation of SCP noted above—that it is mainly about protecting the environment from human activity—is conceptually problematic and adds to the difficulties of getting political traction. A more nuanced framing, which includes also the social dimensions of SCP and focuses on how to enhance and maintain human well-being within ecological constraints, is likely to help to sidestep otherwise insurmountable political hurdles. Even so, creating synergies between ecological sustainability and social progress will require major institutional changes which are likely to be resisted by the beneficiaries of current socioeconomic and political arrangements.

There is a substantial and growing body of scholarship on alternative economies (both within and beyond the capitalist system), but these frameworks are typically extremely marginal. Public policies can play an important role in supporting such pioneering efforts, but progress will likely entail a revitalisation and deepening of democracy. Vast, ambitious, and perhaps very bold political undertaking require partnerships with social movements seeking justice and radical change to the dominant ways in which relationships of production and consumption are structured (Polanyi 1944; Raskin et al. 2002; Wright 2010; Fligstein and McAdam 2015). Many organisations and mobilizations are now actively exploring and testing elements of a new social order beyond consumerism and ecological bankruptcy.^4^ Similarly, private philanthropy and assertions of responsible capitalism are also emerging (Wells 2013; Hoffman 2018). Partnerships among academics, environmental justice campaigners, and labour rights activists for example will in future years be critical to this overall effort and have already begun to coalesce under the umbrella of a notion of “just transitions” (Newell and Mulvaney 2013; Stevis and Felli 2015; Evans and Phelan 2016; Heffron and McCauley 2018).

It is also important to look to the role that cities might play as incubators of scalable and transferable social innovations. Although urban modes of living are often—and not incorrectly—associated with energy- and material-intensive lifestyles, many (but not all) sustainable solutions are pioneered in cities. In the future, it will be necessary to identify urban policies, governance mechanisms, and multi-stakeholder collaborations that address the tensions among conflicting issues and create synergies across various domains and that simultaneously address multiple problems. These interventions will need to include coordinating top–down policy and urban planning processes while enabling innovative citizen-led initiatives that steer sustainability transformations.

## Conclusions and outlook

The main finding of this assessment is the substantial gap that exists between current scientific understanding of sustainable consumption and production (SCP) and how this field is articulated in the 2030 Agenda for Sustainable Development and the SDGs. We find that the need for substantial changes in patterns of consumption and production is well reflected at the headline level of the Agenda, while the specific targets and indicators of the SDG 12 on SCP provide a partial and inadequate conceptualisation of such transformations and do not reflect current scientific knowledge.

Our analysis shows that elements of SCP are part of several of the SDGs, reflecting the cross-cutting nature of SCP as an objectives and a policy approach. These linkages mean that implementing SDG 12 effectively can also help achieve a range of connected goals. While synergies are likely to be found between SCP and most targets across the 2030 Agenda, there is a goal conflict with Target 8.1 on economic (GDP) growth. We are concerned that if governments will focus their efforts on this particular target—GDP growth-driven economic development—SDG12 and other related goals will fall by the wayside.

For implementing agents of the SDGs, both governments and others, to be able to deliver on the commitments of the 2030 Agenda, it is essential to base actions on best available knowledge. In the case of consumption and production, which are the manifestations of highly complex patterns of socioeconomic organisation, this involves adopting a systemic perspective. Such an orientation has a number of implications, some of which are discussed in this paper. For example, the need to complement interventions aimed to enhance efficiency with other measures that limit overall volumes of consumption while safeguarding livelihoods and human well-being. With existing institutional arrangements, developed in an era of continued economic expansion, curtailing consumption would have serious socioeconomic consequences. The 2030 Agenda is based on the assumption that with technological progress, resulting in enhanced efficiency, society will be able to overcome this contradiction—a view that is popular in policy circles but not well supported by science. The solution to this dilemma lies rather in a restructuring of the economic and social arrangements that require endless growth in consumption. Only with such a transformation will it be possible to reconcile objectives that under current arrangements seem to be in conflict. The SDG 12 can help achieving a range of objectives across the 2030 Agenda, but, as it is currently formulated, it is unlikely to inspire the kind of transformation needed for achieving systems of sustainable consumption and production.

One of the great values of the 2030 Agenda is that it creates forums at various levels of society for dialogue on what kind of development is desirable and how society can make this happen. The scientific community has a critical role to play in these processes and we hope that many researchers will be actively engaged. Such involvement in public discourse is especially important in the current era of growing disregard for truth and reason.

## Footnotes

1 Number of publications containing the phrase “sustainable consumption and production” or “sustainable production and consumption”.

2 There are several exceptions to this general characterisation. Especially notable was Indian Prime Minister Indira Gandhi’s presentation at the United Nations Conference on the Human–Environment held in Stockholm in 1972 where she noted “It is an over-simplification to blame all of the world’s problems on increasing population. Countries with but a small fraction of the world population consume the bulk of the world’s production of minerals, fossil fuels, and so on. Thus we see that when it comes to the depletion of natural resources and environmental pollution, the increase of one inhabitant in an affluent country, at his level of living, is equivalent to an increase of many Asians, Africans, or Latin Americans at their current levels of living.” The full transcript of the speech is available at http://lasulawsenvironmental.blogspot.com/2012/07/indira-gandhis-speech-at-stockholm.html. See also Mathiesen (2014) and Caradonna (2014).

3 For a more detailed overview of the developments on the global SCP agenda can be found in Akenji et al. 2017.

4 As points of reference, we acknowledge the contributions made along these lines by the New Economics Foundation, the New Economy Coalition, and the Next System Project.
